# Biogenesis and Function of T Cell-Derived Exosomes

**DOI:** 10.3389/fcell.2016.00084

**Published:** 2016-08-17

**Authors:** Leandro N. Ventimiglia, Miguel A. Alonso

**Affiliations:** Cell Biology and Immunology, Centro de Biología Molecular “Severo Ochoa,” Consejo Superior de Investigaciones Científicas and Universidad Autónoma de MadridMadrid, Spain

**Keywords:** exosomes, multivesicular endosomes, ESCRT complex, tetraspanins, condensed membranes, MAL protein

## Abstract

Exosomes are a particular type of extracellular vesicle, characterized by their endosomal origin as intraluminal vesicles present in large endosomes with a multivesicular structure. After these endosomes fuse with the plasma membrane, exosomes are secreted into the extracellular space. The ability of exosomes to carry and selectively deliver bioactive molecules (e.g., lipids, proteins, and nucleic acids) confers on them the capacity to modulate the activity of receptor cells, even if these cells are located in distant tissues or organs. Since exosomal cargo depends on cell type, a detailed understanding of the mechanisms that regulate the biochemical composition of exosomes is fundamental to a comprehensive view of exosome function. Here, we review the latest advances concerning exosome function and biogenesis in T cells, with particular focus on the mechanism of protein sorting at multivesicular endosomes. Exosomes secreted by specific T-cell subsets can modulate the activity of immune cells, including other T-cell subsets. Ceramide, tetraspanins and MAL have been revealed to be important in exosome biogenesis by T cells. These molecules, therefore, constitute potential molecular targets for artificially modulating exosome production and, hence, the immune response for therapeutic purposes.

## Introduction

Exosomes are nano-sized membranous vesicles (40–100 nm in diameter) that are secreted into the extracellular space by many cell types. The distinctive characteristic of these vesicles is their endosomal origin as intraluminal vesicles (ILV) within multivesicular endosomes (MVE). This origin contrasts with that of other extracellular vesicles of similar size such as shedding vesicles or ectosomes, which are released from the plasma membrane (Kalra et al., 2012; Raposo and Stoorvogel, 2013). The accumulation of “membrane-bound vesicles” in the lumen of MVE as result of the inward invagination of its limiting membrane, as well as the release of “vesicular inclusions” into the extracellular space after the fusion of MVE with the plasma membrane was firstly observed by Philip Stahl's group in 1983 during studies of the traffic of the transferrin receptor in rat reticulocytes (Harding et al., 1983). This finding was consistent with the selective externalization of this receptor in vesicles by maturating sheep reticulocytes observed simultaneously by Rose Johnstone's group (Pan and Johnstone, 1983). A few years later, the term exosomes was coined to refer to the population of vesicles that are released into the extracellular space upon fusion of MVE with the plasma membrane and that can be recovered from the 100,000 × g pellet of cell-free supernatants of cell culture medium or extracellular fluids (Johnstone et al., 1987). Exosomes have been isolated from a great variety of cultured cells and body fluids, including ascite fluid (Andre et al., 2002; Navabi et al., 2005), bronchoalveolar lavage fluid (Admyre et al., 2003), urine (Pisitkun et al., 2004), blood plasma (Caby et al., 2005), synovial fluid (Skriner et al., 2006), breast milk (Admyre et al., 2007), amniotic fluid (Keller et al., 2007), cerebrospinal fluid (Vella et al., 2008), saliva (Ogawa et al., 2008), semen (Poliakov et al., 2009), bile (Masyuk et al., 2010) and nasal mucus (Wu et al., 2015). Within the immune system, exosome secretion has been described in both innate and adaptive immune cells, including B cells (Raposo et al., 1996), dendritic cells (Zitvogel et al., 1998), platelets (Heijnen et al., 1999), mast cells (Skokos et al., 2001), T cells (Blanchard et al., 2002), macrophages (Nguyen et al., 2003) and natural killer cells (Lugini et al., 2012). A major breakthrough in our current understanding of exosome function is the finding that in addition to lipids, proteins and mRNA, exosomes contain miRNA that can be transferred to recipient cells to modulate their activity (Valadi et al., 2007). Therefore, although they were originally described as being a by-product of red cells maturation, it has become evident after 30 years of exosome research that exosomes play an active role as vehicles for intercellular communication by transporting a wide range of bioactive molecules between different cells and tissues (Tkach and Théry, 2016). This role allows exosomes to regulate many physiological activities, including the immune response.

## General mechanisms of exosome biogenesis

Exosomes originate as ILV during the maturation of MVE (Huotari and Helenius, 2011; Hanson and Cashikar, 2012). Depending on the final destination of their ILV, two main kinds of MVE are defined: degradative MVE, which fuse with lysosomes to promote the breakdown of their intraluminal content, and secretory MVE, which fuse with the plasma membrane to release their cargo into the extracellular space. In addition, MVE are implicated in the formation of specialized endosome compartments such as melanosomes in pigment cells and Weibel-Palade bodies in endothelial cells (Marks et al., 2013). Independently of their final destination, ILV formation relies on the recruitment of specific lipids and proteins on the limiting membrane of MVE, and then on their lateral segregation in order to promote the organization of specialized subdomains, from where ILV inward budding occurs.

The protein content of exosome fractions obtained from the 100,000 × g pellet have been characterized in many types of cell. Exosomes contain proteins normally associated with intracellular trafficking such as Rab-family GTPases, annexins, SNAREs, and the endosomal sorting complexes required for transport (ESCRT)-associated proteins tsg101 and Alix. Since the first description of the tetraspanins CD37, CD63, CD81, and CD82 being enriched in exosomes secreted by B-cells (Escola et al., 1998), various members of this extended family of proteins have been found as being enriched in exosomes of different cell origin (Andreu and Yáñez-Mó, 2014). Exosomes have high contents of cholesterol, sphingomyelin, ceramide and ganglioside GM3 (Wubbolts et al., 2003; Subra et al., 2007; Trajkovic et al., 2008; Llorente et al., 2013; Bosque et al., 2016), which are all of them lipids that are concentrated in condensed membrane domains often referred to as membrane rafts (Lingwood and Simons, 2010). Consistent with this observation, exosomes are enriched in proteins associated with these specialized membranes (Wubbolts et al., 2003; Staubach et al., 2009; Dubois et al., 2015), including molecules whose protein moiety is linked to the external leaflet of the membrane by a glycosylphosphatidylinositol anchor, such as CD59 and the enzyme acetylcholinesterase (Johnstone et al., 1987; Rooney et al., 1993; Rabesandratana et al., 1998; De Gassart et al., 2003) or is linked to the internal leaflet by fatty acids, such as Src-family tyrosine kinases (De Gassart et al., 2003), or by proteins with membrane-associated domains, such as caveolin-1 and the membrane tetraspanning MAL protein (Llorente et al., 2004; Ventimiglia et al., 2015).

**Figure 1:**
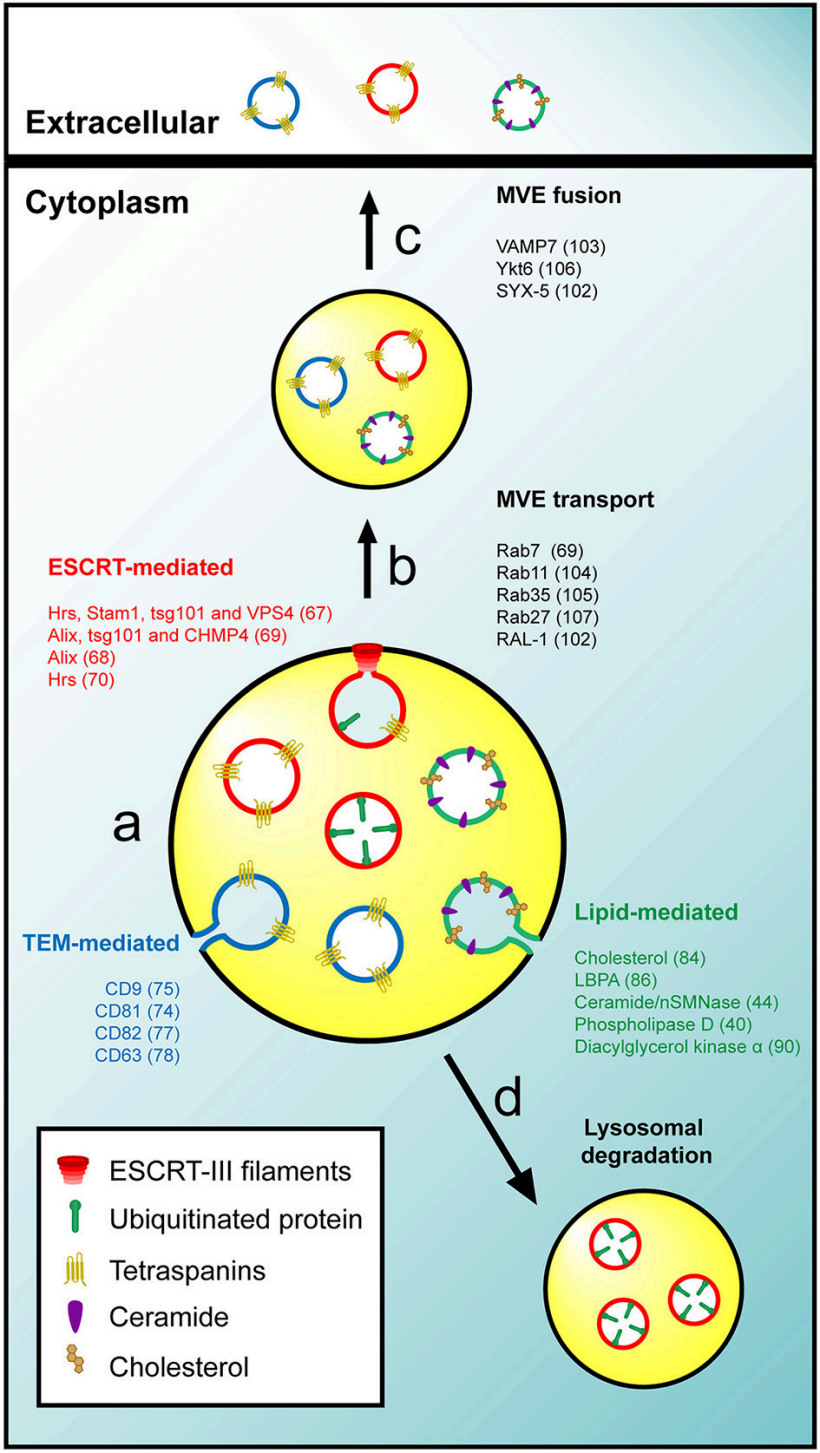
**General mechanisms of exosome biogenesis. (A)** The intraluminal budding of specific membrane nanodomains from the limiting membrane of MVE gives rise to ILV. The sorting of cargo and the invagination of ILV could be mediated by the activity of the ESCRT machinery (red), TEM (blue) or specific lipids such as cholesterol or ceramide (green). **(B,C)** Secretory MVE are transported to the plasma membrane and release their ILV into the extracellular space after membrane fusion. **(D)** The ESCRT-mediated sorting of ubiquitinated proteins gives rise to degradative MVE that fused with lysosomes for degradation of their intraluminal cargo. It is not known whether distinct exosomes and ILV to be secreted as exosomes exist or there is only one class with discrete subdomains. For the sake of simplicity, the three mechanisms were represented in the same MVE, but it remains to be determined whether each mechanism originates a specific kind of MVE or if different populations of ILV coexist in a single MVE. In addition three types of ILV and exosomes are represented although is not clear whether exist different three types of structure or a single structure with discrete subdomains. The molecules involved in the different processes are indicated.

The ESCRT machinery is involved in the formation of ILV in MVE and the recruitment of ubiquitinated proteins to target them for degradation in lysosomes (Babst et al., 2002a,b; Bache et al., 2003). Tetraspanins on one hand, and lipids and proteins associated with condensed membranes on the other, promote segregation of specific membrane subdomains through the organization of protein-protein and protein-lipid interaction networks, respectively. Consistent with the presence in exosomes of ESCRT-associated machinery, tetraspanins and condensed membranes, it has been proposed that distinct molecular mechanisms sort the exosome cargo and deform the membranes required for the inward invagination of ILV destined for secretion as exosomes (Figure 1). However, whether these mechanisms act separately in the same cell to give rise to distinct subpopulations of MVE with a single type of ILV each, converge to generate distinct subsets of ILV in a single type of MVE, or coordinate to generate a single type of ILV structured into discrete membrane domains remains to be clarified. In this regard, evidence exists supporting either possibility. For instance, depending on their activation state, dendritic cells sort MHC II molecules to degradative MVE in a ESCRT-dependent manner or, upon the incorporation of MHC II molecules in tetraspanin-containing condensed membranes, to secretory MVE (Buschow et al., 2009). In addition, it was demonstrated that inactivation of the syndecan-syntenin-Alix axis in breast epithelial MCF-7 cells reduces the release of CD63-positive exosomes, suggesting collaboration between ESCRT and tetraspanins in the biogenesis of secretory MVE (Baietti et al., 2012). It is not clear why different cell types use distinct mechanisms for exosome biogenesis. A possibility is that, whereas most cargos can use any of the mechanisms, certain cargoes require specific machinery to be sorted to ILV destined for exosomes.

### ESCRT-mediated sorting

The ESCRT machinery consists of five protein complexes known as ESCRT-0, -I, -II, -III, and Vps4, and several accessory proteins including Alix (Henne et al., 2011; Schuh and Audhya, 2014). ESCRT components work sequentially in order to sequestrate ubiquitinated proteins in phosphatidylinositol-3-phosphate-enriched domains of the MVE limiting membrane and then facilitate the inward invagination of these domains, promoting the budding and pinching off of ILV. However, the involvement of the ESCRT in the formation of ILV with a secretory destination is less general and seems to be dependent on the cell type and the physiological status of the cells. An shRNA-based screen of 23 ESCRT components in epithelial HeLa cells showed that not all the members of the complex have a role in exosome formation (Colombo et al., 2013). Thus, silencing of the ESCRT-0 members Hrs and Stam1 or the ESCRT-I component tsg101 reduced the release of exosomes, whereas silencing of Vps4 increased it. A role for the accessory protein Alix in sorting the transferrin receptor into exosomes was also observed in erythrocytes (Géminard et al., 2004). In a different study Alix, tsg101, and the ESCRT-III subunits CHMP4A, B, and C were shown to be required for exosome secretion in breast carcinoma MCF-7 cells through a mechanism involving the interaction of Alix with syndecans-syntenin (Baietti et al., 2012). Finally, it was observed that the ESCRT-0 protein Hrs is required for the release of exosomes in dendritic cells (Tamai et al., 2010). On the other hand, a number of studies indicate that ESCRT components are dispensable for exosome secretion, suggesting the existence of ESCRT-independent mechanisms. For instance, the silencing of Hrs, tsg101, and Alix, as well as the overexpression of a dominant negative mutant of Vps4 had no effect on intraluminal sorting or exosomal release of the proteolipid protein in mouse oligodendroglial Oli-neu cells (Trajkovic et al., 2008). Hrs was also shown to be dispensable for sorting of the pigment cell-specific protein 17 into melanosomal ILV in MNT-1 melanoma cells (Theos et al., 2006).

### Tetraspanin-mediated sorting

The tetraspanin protein family consists of 33 members in humans. Tetraspanins have low sequence homology but are similarly structured in four transmembrane domains with both amino- and carboxyl-termini of the molecule oriented toward the cytoplasm. Tetraspanins interact with themselves and with other membrane and cytosolic proteins to organize a particular kind of membrane domains known as tetraspanin-enriched microdomains (TEM). Tetraspanins mediate a number of cellular processes including cell adhesion, immune activation, tumor and immune cell extravasation, virus entry and intracellular trafficking regulation (Yáñez et al., 2009; Charrin et al., 2014). TEM appear to play a role in sorting the intraluminal cargo and the secretion of exosomes. In dendritic cells, the loading of MHC-II in exosomes depends on its incorporation into membrane domains enriched in the tetraspanin CD9 (Buschow et al., 2009), and this protein was also shown to be required for the incorporation of the metalloprotease CD10 into exosomes of erythroleukemic K562 cells (Mazurov et al., 2013). Bone marrow dendritic cells derived from CD9 knockout mice show reduced secretion of exosomes, while the overexpression of tetraspanins CD9 or CD82 in human embryonic kidney 293 cells increases the incorporation of β-catenin in exosomes (Chairoungdua et al., 2010). In melanocytes the sorting of the luminal domain of pigment cell-specific protein 17 also depends on the expression of CD63 to be included into ILV during melanosome formation (van Niel et al., 2011).

### Lipid-mediated sorting

The ability of lipids to laterally segregate and self-organize into specific membrane subdomains allows sorting platforms to be organized on cell membranes, including the limiting membrane of MVE (Bissig and Gruenberg, 2013). Accordingly, a subset of lipids and lipid-metabolizing enzymes has been implicated in the sorting of cargo into ILV as well as in exosome release. Cholesterol is a sterol lipid that plays a key role in organizing the biophysical properties of cell membranes (Maxfield and van Meer, 2010). Cholesterol was shown to be necessary for the formation of highly curved membrane structures such as caveolae (Chang et al., 1992) and synaptic vesicles (Thiele et al., 2000). Since cholesterol is highly enriched in ILV of MVE but poorly represented in lysosomes, a role for cholesterol was proposed in the intraluminal sorting of membranes destined for secretion as exosomes (Möbius et al., 2003). Lysobisphosphatidic acid (LBPA) is a coned-shaped lipid that is also abundant in internal membranes of MVE (Kobayashi et al., 1998). LBPA controls the formation of ILV both *in vitro* and *in vivo* through the recruitment of Alix (Matsuo et al., 2004). However, since LBPA mostly concentrates in lysosomes and is present in only small amounts in exosomes, it was proposed that LBPA is not involved in the formation of ILV with a secretory destination but rather in backfusion of ILV with the MVE limiting membrane (Bissig and Gruenberg, 2014). Ceramide, another coned-shaped lipid, promotes the coalescence of membrane domains and induces the spontaneous curvature of membranes (Castro et al., 2014). The activity of neutral sphingomyelinase (nSMNase) II, which is the enzyme that catalyzes the synthesis of ceramide from sphingomyelin, is required for the inward budding of proteolipid protein-bearing ILV as well as for its secretion in exosomes in oligodendroglial Oli-neu cells, while it is not involved in the luminal sorting of epidermal growth factor receptor (Trajkovic et al., 2008), which follows the degradative pathway. Phospholipase D, which is required for the secretion of exosomes in rat basophilic leukemia 2H3 cells (Laulagnier et al., 2004), is one more example of lipid-metabolizing enzyme involved in exosome biogenesis.

## Biogenesis of exosomes by T cells

There is little information regarding the cellular machinery that controls exosome biogenesis in T cells. In exosomes from human lymphoblasts, tetraspanins and their associated proteins form a network of interactions that comprise 45% of the total protein content (Perez-Hernandez et al., 2013). In mice deficient in tetraspanin CD81, these exosomes have a reduced content of CD81-associated proteins, such as B-cell receptor, CD20, ICAM-1, and HLA isotypes (Perez-Hernandez et al., 2013). These observations indicate a role for TEM in sorting of specific proteins to ILV destined for exosome secretion. The association of the oncogene latent membrane protein 1 of Epstein Barr virus with tetraspanin CD63-enriched microdomains is required for its sorting into ILV and its secretion via B cell exosomes, as demonstrated by CD63 silencing (Verweij et al., 2011). Although the evidence is still preliminary, the role of tetraspanins in protein sorting to ILV and their ubiquitous presence in exosomes make it plausible that tetraspanins, and subsequently TEM, also play a role in exosome biogenesis in T cells. As occurs in Oli-neu cells (Trajkovic et al., 2008), ceramide is important for the formation of ILV destined for exosomes in T cells, as silencing of nSMNase II or inhibition of its enzymatic activity induce a decrease in the release of exosomes (Mittelbrunn et al., 2011; Ventimiglia et al., 2015). Some lipid-metabolizing enzymes, such as diacylglycerol kinase α, accumulate on the limiting membrane of T cell MVE and control the maturation of MVE and the release of CD63-containing exosomes in T and B cells (Alonso et al., 2011), reinforcing the proposed role of lipids in exosome formation by T cells. A role for condensed membranes in exosome formation by T cells is further supported by the observation that T cell-derived-exosomes are enriched in sphingomyelin and cholesterol, as observed in a recent lipidomic analysis (Bosque et al., 2016). The observations that neither Hrs nor tsg101 are required for exosome release by T cells (Mittelbrunn et al., 2011; Ventimiglia et al., 2015) indicate that, although other ESCRT components have not been tested yet, exosome biogenesis appears to be independent of ESCRT machinery unlike in other cell types (Géminard et al., 2004; Baietti et al., 2012; Colombo et al., 2013).

MAL is a 17-kDa tetraspanning membrane protein whose expression is restricted to T cells, specific polarized epithelia and myelin-forming cells (Alonso and Weissman, 1987). A characteristic biochemical feature of MAL is its exclusive association with detergent-insoluble membrane fractions enriched in condensed membranes (Millán and Alonso, 1998). MAL is essential for the transport of the tyrosine kinase Lck to the plasma membrane of T cells, and controls the condensation of membranes at the immunological synapse, ensuring the correct sorting of Lck and LAT into this structure (Antón et al., 2008, 2011). The membrane trafficking processes characterized so far are dependent on protein machinery. Lipids collaborate in these processes by helping to bend membranes to form transport vesicles (e.g., ceramide, LBPA) or by providing sites for recruiting specific protein machinery (e.g., phosphoinositides). An important clue about the cellular machinery involved in sorting of cargo and the biogenesis of exosomes in T cells, as well as about the mechanisms that regulate the collaboration between TEM and condensed membranes comes from the study of the role of the protein MAL during MVE maturation and the secretion of exosomes in T cells (Ventimiglia et al., 2015). MAL distributes in tubular-vesicular structures characteristic of early endosomes, some of which present MVE morphology. The silencing of MAL in T cells reduces the number of exosomes secreted, and subsequently the release of the exosome cargo. This effect was correlated with reduced sorting of CD63 into the intraluminal space of MVE and its accumulation on the limiting membrane of aberrant MVE that no longer fuse with the plasma membrane but, instead, merge with autophagic vacuoles and lysosomes. Therefore, the protein MAL appears as a key component of the machinery responsible for exosome biogenesis and sorting of cargo, including tetraspanins, destined for exosome delivery in T cells. The role of MAL in Lck trafficking, organization of the immunological synapse and exosome biogenesis is probably related to the capacity of MAL to organize condensed membrane domains (Antón et al., 2008, 2011; Magal et al., 2009).

**Figure 2:**
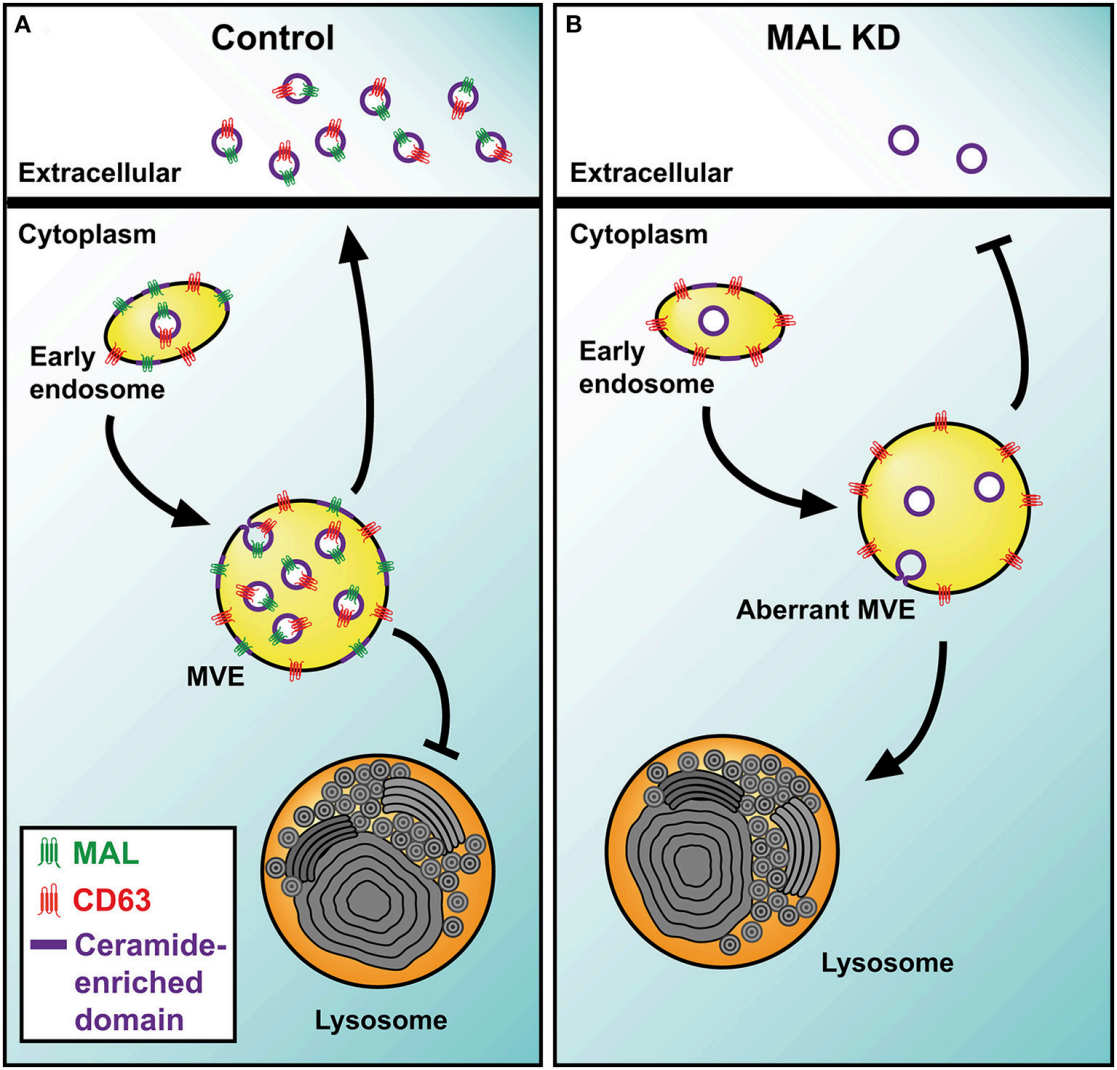
**Model of MAL function in exosome biogenesis by T cells. (A)** The transmembrane protein MAL facilitates the incorporation of TEM into ceramide-enriched condensed nanodomains at the limiting membrane of MVE. The intraluminal budding of the TEM-condensed mixed nanodomains gives rise to the ILV that are released as exosomes. Although MAL also intervenes in other membrane trafficking processes, only those related to exosome biogenesis are represented. **(B)** In MAL-silenced T cells, TEM are not incorporated efficiently into ILV and accumulate on the limiting membrane of MVE. These aberrant MVE are not able to fuse with the plasma membrane and divert to lysosomes for degradation.

From a biochemical point of view exosomes could be considered to be specialized membranes highly enriched in TEM and condensed domains. Although these two distinctive membrane domains are biochemically and functionally distinguishable entities (Le Naour et al., 2006), it has been demonstrated that the progression of many cellular processes rely on the ability of TEM and condensed membranes to cooperate in order to build functional membrane scaffolds. There are a number of examples that illustrate a relationship between condensed membranes and TEM that are consistent with a possible joint role of these two types of membrane in exosome biogenesis. For example, the incorporation of the B-cell receptor into condensed membranes, a key step to ensure the sustainment and the amplification of B-cell receptor-mediated signaling, depends on the expression of CD81 (Cherukuri et al., 2004); in T cells the tetraspanin CD82 mediates the association of the actin cytoskeleton with compact membranes, which is necessary for triggering T-cell receptor-dependent signaling (Delaguillaumie et al., 2004); the incorporation of CD9 into condensed membranes domains is crucial for the cell fusion during osteoclastogenesis (Ishii et al., 2006); and the recruitment of CD63 to filopodia tips during the adhesion of platelets depends on the membrane compaction (Heijnen et al., 2003). In this regard, the cooperation between TEM and condensed membranes could have a key importance during the biogenesis of exosomes, firstly mediating the organization of differentiated domains on the limiting membrane of MVE through the recruitment of specific exosome components, and then, promoting the invagination of these domains to form ILV. Supporting a collaboration between condensed membranes and TEM, it was reported that β-catenin secretion in exosomes of bone marrow-derived dendritic cells is impaired in CD9 knockout mice and also by nSMNase II inhibition (Chairoungdua et al., 2010). Despite these advances, the mechanisms that regulate the interaction between TEM and condensed membranes require further investigations. Given the capacity of MAL to organize condensed membrane domains (Antón et al., 2008, 2011; Magal et al., 2009), the presence of ceramide in this type of membrane and the dependence of the exosome secretion of CD63 on both ceramide and MAL expression, it is plausible that the correct intraluminal sorting of CD63 to secretory MVE requires MAL-organized ceramide patches at the MVE limiting membrane to promote cargo selection and the inward invagination of the patches leading to the formation of ILV for exosome secretion (Figure 2).

## Function of T cell-derived exosomes

Although a large number of proteins are commonly found in exosomes regardless of their cellular origin (Choi et al., 2015), the protein composition of exosomes varies with cell type, somehow reflecting the specific functions of the producer cell. For instance, exosomes derived from B cells carry B-cell receptor subunits (Rialland et al., 2006) and those released by natural killer cells contain the cytolitic protein perforin and CD56 (Lugini et al., 2012), which is a marker of this type of cell. Consistent with this correlation, normal T cells secrete exosomes containing T-cell receptor subunits, Src-like tyrosine kinases and adhesion molecules (Blanchard et al., 2002).

With regard to the function of exosomes secreted by T cells, diacylglycerol kinase α inhibition is known to increase the secretion of lethal exosomes bearing membrane-associated Fas ligand to mediate activation-induced cell death in other T cells (Alonso et al., 2005). The exosomes derived from stimulated human CD3^+^ T cells cooperate with IL2 in promoting proliferation of autologous resting cells (Wahlgren et al., 2012). T-cell tolerance of allergic cutaneous contact sensitivity induced in mice is shown due to exosomes secreted by CD8+ suppressor T cells (Bryniarski et al., 2013). Also, in mice, Fas ligand-containing exosomes secreted by activated T cells promote tumor invasion in lungs by increasing the expression of matrix metalloproteinase 9 (Cai et al., 2012), and CD8^+^CD45^+^ regulatory T cell-released exosomes inhibit CD8^+^ cytotoxic T-lymphocyte response and antitumor activity (Zhang et al., 2011). The exosomes secreted by CD4^+^ CD25^+^ regulatory T cells prolong the survival time of kidney transplants and inhibit T cell proliferation in rat models, indicating that exosomes contribute to transplantation tolerance (Yu et al., 2013).

Similar to the case of exosomes from other sources (Valadi et al., 2007; Kosaka et al., 2010), exosomes secreted by human T cells deliver biologically active miRNA (Mittelbrunn et al., 2011). miRNA can be unidirectionally transferred from T cells to antigen-presenting cells through the immunological synapse using exosomes as vehicles (Mittelbrunn et al., 2011). The importance of exosome miRNA in modulating the immune response is revealed by the observation that Let-7d miRNA, which is found in exosomes derived from Foxp3^+^ T regulatory cells, suppresses T helper 1 cells proliferation and γ-IFN secretion (Okoye et al., 2014).

In addition to their role in controlling the immune response, exosomes play a role in pathogenesis, with some pathogens being able to modulate the secretion of exosomes in the host to favor its own propagation. For example, the expression of the HIV-1 protein Nef induces the secretion of exosomes either in HIV-1 infected or in Nef-transfected T cells (Campbell et al., 2008; Muratori et al., 2009; Lenassi et al., 2010; Raymond et al., 2011; Shelton et al., 2012; Lee et al., 2013). It has been shown that HIV-1 usurps the exosome-based intercellular communication network of T cells to render quiescent T cells permissive to HIV-1 replication (Arenaccio et al., 2014a,b) promoting their apoptosis (Lenassi et al., 2010) and stimulating an extensive secretory activity leading to the massive release of microvesicles (Muratori et al., 2009).

## Conclusions

Exosomes secreted by T cells have emerged as key mediators of the immune response, modulating the activity of immune cells. Modulation of the exosome route in T cells, therefore, may be of therapeutic value for preventing T cell-mediated diseases such as inflammation or transplant rejection or for interfering with the progression of HIV-1 infection (Agarwal et al., 2014; Zhang et al., 2014; De Toro et al., 2015; Liu et al., 2015; Sáenz-Cuesta et al., 2015). Ceramide, tetraspanins and MAL, which have been revealed to be important in exosome biogenesis by T cells, are candidate molecular targets for artificially modulating exosome production by T cells. A better understanding of the basic molecular mechanisms that regulate the release of exosomes in these cells becomes fundamental not only to gain more insight into the roles of these vesicles during the onset and progression of a robust immune response, but also to enable the design of engineered exosomes for use as therapeutic agents.

## Author contributions

LV and MA wrote the Review. LV designed the Figures. MA supervised everything.

## Funding

Research in the laboratory of MA is supported by grants (BFU2012-32532 and BFU2015-67266-R) from the Ministerio de Economía y Competitividad and Fondo Europeo de Desarrollo Regional (MINECO/FEDER).

### Conflict of interest statement

The authors declare that the research was conducted in the absence of any commercial or financial relationships that could be construed as a potential conflict of interest.

## Abbreviations

ESCRT: endosomal sorting complexes required for transport
ILV: intraluminal vesicle(s)
LBPA: lysobisphosphatidic acid
MVE: multivesicular endosome(s)
nSMNase II: neutral sphingomyelinase II
TEM: tetraspanin-enriched microdomain(s).
